# Retrospective Study of Pediatric Hip Dislocations and Proposal of a Novel Classification System for Risk Assessment

**DOI:** 10.7759/cureus.117882

**Published:** 2026-10-13

**Authors:** Saksham Pruthi, Vinieth Bijanki, Sivashanmugam Raju

**Affiliations:** 1 Department of Orthopaedic Surgery, Saint Louis University School of Medicine, Saint Louis, USA

**Keywords:** classification system, closed fracture reduction, fracture around hip, hip dislocation, pediatrics

## Abstract

Introduction: Traumatic hip dislocations in children are rare but carry high morbidity due to risks such as avascular necrosis (AVN) and post-traumatic arthritis. Existing classification systems are designed for adults and fail to account for pediatric-specific factors, such as ongoing skeletal maturation. This study aimed to characterize pediatric hip dislocations from a single level I trauma center, evaluate associated complication risks, and propose a novel classification system for the pediatric population.

Materials and methods: We conducted a retrospective cohort study of patients aged 2-18 years with traumatic hip dislocations treated between January 2015 and January 2025 at a single level I pediatric trauma center. Demographics, mechanism of injury, associated injuries, reduction details, and outcomes were all analyzed. Patients were categorized into four proposed types based on mechanism, time to reduction, method/location of reduction, and associated injuries. Statistical analysis included descriptive statistics, chi-squared or Fisher’s exact tests, and ANOVA with significance set at p < 0.05.

Results: Thirty-four patients met the inclusion criteria. The mean age of patients was 13.7 ± 3.4 years, with a mean follow-up of 13.9 months (minimum six months). We found that posterior acetabular fractures were significantly more common in high-energy injuries than in falls (p = 0.025). Our institution’s median time to reduction was 6.5 hours, with no significant association between time to reduction and development of complications like AVN or arthrofibrosis. Classification type was significantly associated with skeletal maturity (p = 0.049), whereas no significant association was observed with BMI (p = 0.749).

Conclusions: Pediatric hip dislocations demonstrate distinct injury patterns influenced by maturity, BMI, and mechanism. Our proposed classification system provides a pediatric-specific framework for describing injury patterns and treatment complexity. Further prospective and external validation is required to determine whether classification type has predictive value for clinical outcomes or management.

## Introduction

Traumatic hip dislocations in children are relatively rare but carry significant clinical consequences due to the risk of significant complications, especially avascular necrosis (AVN). While the incidence of traumatic pediatric hip dislocations is reported to be less than 5% of all hip dislocations, the majority of these are posterior dislocations [1]. These injuries often result from high-energy mechanisms, including motor vehicle collisions or falls from height, particularly in older children and adolescents. In younger children, even relatively low-energy trauma is sufficient to cause dislocations due to underdeveloped acetabular anatomy and increased joint laxity [2].

Timely reduction of the dislocated hip is considered the cornerstone of treatment. Prior studies have demonstrated a direct correlation between delayed reduction (more than six hours post-injury) and increased rates of AVN [1-3]. Mehlman et al. found that the AVN rate was 20 times higher in patients whose time from injury to reduction exceeded the six-hour window [3].

Despite this, delays in diagnosis and reduction remain common, often due to missed injuries in polytrauma settings or lack of early imaging [3,4]. Moreover, the literature remains sparse regarding long-term outcomes following traumatic hip dislocations in children, especially with respect to the influence of demographic and injury-related variables. In addition to AVN, other complications such as post-traumatic arthritis, arthrofibrosis, and chondrolysis are possible and can result in significant long-term disability [4]. The pathophysiology of these complications is multifactorial, potentially related to initial trauma severity, time to reduction, associated injuries, and reduction techniques.

Associated injuries-such as acetabular fractures, femoral head injuries, and labral tears-also contribute to prognosis and often necessitate surgical intervention. These concurrent injuries are more common with high-energy mechanisms and may increase the risk of post-reduction complications.

Several classification systems exist for adult hip dislocations, but these are not directly applicable to pediatric populations due to differences in anatomy and biomechanics. Furthermore, given the scarcity of this injury, there remains a need for pediatric-specific classification tools to guide management and facilitate prognostication when they do occur. Recent efforts in orthopedic trauma literature suggest that a combination of clinical, radiographic, and intraoperative variables could inform a novel classification approach. Such a system would help stratify injury severity and predict outcomes, aiding clinical decision-making.

The primary objective of this retrospective study was to characterize the clinical presentation, injury patterns, management, and complications of traumatic hip dislocations in a pediatric population. Secondary objectives were to (1) evaluate the association between patient and injury characteristics and post-reduction complications and (2) develop a pediatric-specific classification framework based on mechanism of injury, associated fractures, reduction timing, and reduction method/location. We additionally evaluated whether skeletal maturity and body mass index (BMI) percentile were associated with classification type. Given the rarity of pediatric hip dislocations, this classification was intended as a descriptive and hypothesis-generating framework to facilitate characterization of injury complexity and guide future validation studies.

## Materials and methods

This retrospective cohort study evaluated patients aged 2-18 years with traumatic hip dislocations treated at our level I pediatric trauma center, from January 1, 2015, through January 1, 2025. Inclusion criteria for our study included patients between the ages of two and 18 who had a traumatic dislocation that was reduced at our institution (either in the emergency department (ED) or the operating room) and had at least six months of follow-up. Follow-up was defined as any clinical encounter with a pediatric orthopedic surgeon or a primary care physician that specifically addressed hip dislocation. Exclusion criteria included non-traumatic etiologies, incomplete documentation, follow-up < six months, and transferred cases with missing outside data. Using these specific criteria, we queried the electronic medical record (EMR) in Epic’s Slicer Dicer tool (Epic Systems Corporation, Verona, WI, USA) to identify potential participants and manually screened them for final inclusion in the dataset.

The initial query search yielded 90 patients. After review, we excluded 29 individuals who presented with concerns of hip dislocation but were found to have a different pathology responsible for their hip pain. We then had another 23 patients present with hip dislocations due to atraumatic etiologies, such as cerebral palsy, which were excluded from our dataset. Lastly, we removed four patients who were transferred from outside hospitals and were missing either imaging or information about time to reduction and mode of injury. This brought us to the final 34 patients included in our dataset for analysis and synthesis of our classification system.

Following identification of the final cohort, data were manually extracted from the EMR using a standardized data collection process by members of the research team appointed and trained by the principal investigator. Demographic variables included age, sex, race, and BMI percentile. Injury-related variables included mechanism of injury, direction of dislocation, associated acetabular or femoral head fractures, time from injury to reduction, reduction method, and reduction location. Post-reduction outcomes included AVN, post-traumatic arthritis, arthrofibrosis, infection, retained loose bodies, and need for additional surgery. Data were obtained from ED and operative notes, radiology reports, clinic notes, and follow-up imaging when available. A threshold of six hours was specifically selected for time to reduction based on the work of Mehlman et al., who demonstrated a substantially increased risk of AVN when reduction was delayed beyond six hours after injury [3]. Because of the established prognostic significance of this variable, reduction timing was incorporated directly into the classification system as a determinant of injury complexity rather than analyzed solely as an independent outcome measure. Reduction was classified by method (closed versus open) and location (ED versus operating room). These variables were not permitted to override the primary mechanism/fracture-based classification assignment. Accordingly, data were abstracted for a minimum of six months following the initial presentation to capture the frequency of these complications.

To develop a classification system for this study, we looked at variables relevant to injury severity. The variables selected for inclusion in the classification framework were chosen based on factors consistently reported in the literature to influence injury severity, treatment complexity, and risk of complications following pediatric traumatic hip dislocation. Mechanism of injury (fall, high-energy trauma, etc.) was included because high-energy trauma is frequently associated with greater soft tissue damage and a higher incidence of concomitant injuries [1,2]. The presence of associated fractures (acetabular, femoral head, or both) was included as a marker of injury complexity and because acetabular and femoral head fractures often necessitate additional surgical management [5]. Method (closed versus open) and location (emergency room (ER) versus operating room) of reduction were selected because the need for operative intervention or reduction under anesthesia may reflect increased injury severity, difficulty of reduction, or the presence of associated pathology. Time to reduction (hours) was incorporated because delayed reduction has been identified as one of the most important predictors of adverse outcomes, particularly AVN, in previous studies [4-6]. Using these factors, patients were then assigned to one of four categories using a decision-based framework: Type I: Low-Energy, Isolated: defined as dislocations due to falls with no associated fractures, often managed with closed reduction, and time to reduction is usually under six hours. Type II: Low-Energy, Complex: also due to falls but occur with associated fractures, often managed with open reduction and/or delayed reduction in the ER (≥6 hours). Type III: High-Energy, Isolated: involving traumatic mechanisms without associated fractures and often managed with closed reduction within six hours. Type IV: High-Energy, Complex: characterized by trauma with associated fractures, usually associated with delayed ER reduction or reduction in the operating room.

To explore potential patterns in classification distribution, patients in the proposed classification group were compared by Risser stage (0-5, based on the degree of ossification and fusion of the iliac apophysis, reflecting skeletal maturity: stage 0 = no ossification; stage 1 = up to 25% ossification; stage 2 = 26%-50%; stage 3 = 51%-75%; stage 4 = 76%-100% ossification; stage 5 = complete fusion of the iliac apophysis to the iliac crest) and BMI categories (underweight: <5th percentile, normal: 5th to 85th percentile, overweight: 86th to 95th percentile, and obese: ≥95th percentile).

Statistical analysis was conducted to evaluate the association between patient demographics, time to reduction, injury characteristics, and clinical outcomes, including AVN, arthrofibrosis, osteoarthritis, infection, and need for reoperation. Descriptive statistics were used to summarize patient demographics, injury characteristics, and classification distributions. Categorical variables, such as postoperative complications and sites of injury, were compared using chi-squared or Fisher’s exact test (due to small sample sizes), and continuous variables were assessed with independent t-tests or ANOVA where appropriate. All statistical analyses were performed in Microsoft Excel Version 16.99 (Microsoft Corp., Redmond, WA), with statistical significance set at p < 0.05. BMI percentile calculations were done using the CDC values for age, sex, height, and weight.

## Results

For the 34 patients included in this study, the average age was 13.67 (±3.40). The average follow-up time for these patients was 13.9 months (6-56 months). The demographic data are summarized in Table 1.

**Table 1:** Patient demographics. BMI: body mass index

Variable	Count
Participants (N)	34
Age (mean ± SD)	13.67 ± 3.40
Sex	
Male (%)	65%
Female (%)	35%
Race (N, %)	
White	17 (50%)
African American	13 (38%)
Hispanic	1 (3%)
Asian	1 (3%)
Other	2 (6%)
BMI percentile (mean ± SD)	76.45 ± 26.42
Mode of injury (N, %)	
Fall	9 (26%)
Trauma	25 (74%)
Dislocation direction (N, %)	
Posterior	31 (92%)
Anterior	3 (8%)

The associated injuries that patients experienced were grouped based on mechanism of injury, either fall or high-energy trauma such as a motor vehicle collision or assault (Table 2). There were 25 patients who presented with true hip dislocations due to high-energy trauma, with only nine occurring due to low-energy trauma, such as a fall. Among these patients, the most commonly associated injury was a posterior acetabular fracture. Fisher’s exact test analysis was done, which showed there were significantly more posterior acetabular fractures with traumatic hip dislocations when compared to hip dislocations due to falls (p = 0.0252). Anterior acetabular fractures and femoral head fractures were also seen, but there was no significant association between these injury sites and mechanisms. There were also two patients with ipsilateral tibia fractures (one in the fall group and one in the trauma group) that were not included, as they are not commonly associated with hip dislocations.

**Table 2:** Mechanism and associated injuries. p-value < 0.05 is significant (*) using Fisher's exact test.

Mode	No other injuries	Posterior acetabulum	Anterior acetabulum	Femoral head
Fall	4	2	1	1
Motor vehicle accident	5	17	1	1
Test statistic	Fisher's exact test	Fisher's exact test	Fisher's exact test	Fisher's exact test
p-value	0.2009	0.0252*	0.4652	0.4652

Of the 34 patients, 33 had their dislocations concentrically reduced in the ED, with only one requiring closed reduction under anesthesia in the operating room. Of those 34 patients, the vast majority (92%) had hips dislocated posteriorly. Patients who had associated fractures, such as of the posterior acetabulum or femoral head, were subsequently treated operatively for those associated injuries after being reduced. Table 2 presents the most common associated injuries for all 34 patients, and Table 3 provides a breakdown of the specific surgical interventions for the 25 patients with concurrent injuries. Postoperative recommendations for all patients with hip dislocations and a concurrent posterior acetabular fracture were to be non-weight-bearing with posterior hip precautions.

**Table 3:** Breakdown of surgical management.

Surgery	N (%)
Acetabular open reduction and internal fixation	16 (64%)
Femoral head open reduction and internal fixation	1 (4%)
No surgery	8 (32%)

We also examined the time to reduction to assess its effect on complication incidence. We had 15 patients reduced in less than six hours, and 19 reduced at six hours or longer from injury. In our dataset, the median time to reduction from injury was 6.5 hours. Given how close this was to the six-hour threshold set by Mehlman et al. [3], we studied the frequency of AVN, post-dislocation arthritis, arthrofibrosis, infection, and retained loose bodies in our patients using this six-hour cut-off. We assessed the rate of these complications by reading the radiographs and clinic notes from the patients’ outpatient follow-ups post-reduction. Using Fisher’s exact test for analysis, we found no difference in time to reduction and development of complications (Table 4). The same variables with patients who had at least one year of follow-up are shown in Table 5.

**Table 4:** Post-reduction complications with a 6-hour threshold to reduction. p-value < 0.05 is significant (*) using Fisher's exact test. AVN: avascular necrosis

Time to reduction	N	AVN	Arthritis	Arthrofibrosis	Infection	Retained loose body
<6 hours	15	2	0	1	1	0
>6 hours	19	1	1	0	0	1
Test statistic	Fisher’s exact test	Fisher’s exact test	Fisher’s exact test	Fisher’s exact test	Fisher’s exact test	Fisher’s exact test
p-value	-	0.555	0.4118	0.99	0.4118	0.99

**Table 5:** Post-reduction complications with a 6-hour threshold to reduction in patients with a minimum 1-year follow-up. p-value < 0.05 is significant (*) using Fisher's exact test. AVN: avascular necrosis

Time to reduction	N	AVN	Arthritis	Arthrofibrosis	Infection	Retained loose body
<6 hours	8	1	0	0	1	0
>6 hours	12	1	0	0	0	1
Test statistic	Fisher’s exact test	Fisher’s exact test	Fisher’s exact test	Fisher’s exact test	Fisher’s exact test	Fisher’s exact test
p-value	-	1.0	1.0	1.0	0.4	1

We finally used the classification method we had proposed to group the patients included in this study, stratifying them by BMI (Table 6) and skeletal maturity (Table 7). Using Fisher’s exact test, we found a p-value of 0.049 when group placement was significantly compared with skeletal maturity. The p-value was 0.749 when comparing group placement and BMI. 

**Table 6:** Grouping patients into proposed classification systems by BMI. BMI: body mass index

BMI group	Type I	Type II	Type III	Type IV
<5th percentile	3	2	0	2
5th-85th percentile	2	9	2	0
86th-95th percentile	1	4	1	1
≥95th percentile	1	5	1	0

**Table 7:** Grouping patients into proposed classification systems by Risser stage.

Risser stage	Type I	Type II	Type III	Type IV
0	1	0	0	0
1	2	2	1	0
2	1	2	2	1
3	2	5	1	1
4	0	2	4	1
5	1	0	4	1

## Discussion

This retrospective examination of pediatric traumatic hip dislocations contributes to the limited body of literature on this rare but clinically significant injury. Our analysis spanned a decade and sought not only to characterize associated risk factors and outcomes but also to propose a novel classification system tailored to the pediatric population. The results highlight several important findings relevant to both clinical management and future research.

Importantly, our proposed classification system also has practical implications for assisting with treatment planning and prognostication. In our cohort, lower-grade injuries (Types I and III) generally reflected isolated, low-complexity dislocations that could be safely managed with prompt closed reduction and early mobilization once stability was confirmed. In contrast, higher-grade injuries (Types II and IV) were more likely to involve associated acetabular or femoral head fractures, open management, and prolonged protected weight-bearing patterns that directly informed surgical decision-making and rehabilitation protocols. As such, the classification can serve as a treatment guide by linking injury severity to the anticipated need for operative intervention, the duration of immobilization, and the intensity of follow-up imaging. Moreover, the presence of complex features within these higher-grade groups may predict an increased risk of long-term complications, such as AVN or arthrofibrosis, enabling clinicians to identify and monitor high-risk patients earlier in their recovery [6-8].

Our classification system stratified patients into four categories based on mechanism of injury, presence of associated injuries, time to reduction, and method/location of reduction. The system allowed clear categorization of injury severity and treatment complexity across demographic subgroups. While adult-based classification systems such as Thompson-Epstein and Pipkin have historically guided the management of traumatic hip dislocations, their applicability to pediatric populations is limited [9-13]. The Thompson-Epstein classification primarily focuses on posterior hip dislocations and associated fracture patterns, whereas Pipkin was mostly only for femoral head fractures. Furthermore, both were developed in adult populations and do not account for pediatric-specific factors such as skeletal immaturity, body habitus, or differences in treatment pathways. In contrast, our classification system incorporates injury characteristics relevant to pediatric patients, including mechanism of injury, associated fractures, reduction timing, and reduction method/location. Patient factors such as BMI and skeletal maturity were subsequently evaluated to determine whether they were associated with classification type rather than being used as classification-defining variables. A comparison of these systems is provided in Table 8. By integrating patient-specific considerations, our system may provide a useful framework for future prospective validation and standardization of pediatric traumatic hip dislocation management.

**Table 8:** Summary of hip classification syndromes. BMI: body mass index

Classification system	Primary focus	Pediatric applicability	Strengths	Limitations
Thompson-Epstein	Posterior hip dislocation type and associated fractures	Limited	Widely recognized; guides adult treatment	Developed for adults; does not consider skeletal maturity, reduction timing, or patient factors
Pipkin	Femoral head fractures associated with hip dislocation	Limited	Useful for fracture characterization	Applicable only when femoral head fractures are present; adult-based
Proposed pediatric classification	Mechanism, associated injuries for both posterior and anterior dislocations in pediatric population	High	Incorporates pediatric-specific factors such as age and BMI; reflects treatment complexity and outcomes	Requires prospective validation and external reproducibility studies

Interestingly, our results did not demonstrate a statistically significant difference in post-reduction complications (including AVN, arthrofibrosis, infection, or retained loose bodies) when stratified by time to reduction, despite previous studies such as Mehlman et al. reporting a 20-fold increased risk of AVN with delays beyond six hours [3]. However, it is important to contextualize this finding within the broader pediatric literature, which consistently supports hip dislocation as an orthopedic emergency requiring urgent reduction. Prior pediatric series and reviews emphasize that reduction should be performed as early as possible, often immediately upon presentation, as delayed management has historically been associated with increased rates of AVN and poorer functional outcomes [14,15]. Rather, our findings align more closely with recent work that observed how factors such as initial trauma severity and presence of femoral head lesions may be more predictive of AVN than time to reduction alone [12]. Similarly, other articles report excellent functional outcomes and a very low incidence of osteonecrosis in a contemporary pediatric cohort treated with prompt reduction, further suggesting that outcomes may depend on a multifactorial interplay among injury severity, associated fractures, and reduction quality rather than on timing alone [16]. This suggests that while early reduction remains a critical goal, it may not be the sole determinant of outcome, particularly when proper anatomical reduction is achieved within a reasonable timeframe.

Importantly, many of these earlier pediatric studies are limited by relatively small sample sizes, often representing single-center series or case-based cohorts [14,15]. In contrast, while our study remains modest in absolute numbers due to the inherent rarity of pediatric hip dislocations, it spans a longer observational period and includes a comparatively larger cohort than many previously published reports. This provides a broader characterization of injury patterns and management variability within this uncommon injury spectrum. Although our sample size still limits statistical power and generalizability, the rarity of pediatric hip dislocation and the heterogeneity in reported management strategies across the literature underscore the value of a structured classification framework to improve consistency in reporting, risk stratification, and future comparative studies.

As it pertains to associated injuries with these pediatric dislocations, our findings align rather closely with prior research. Posterior acetabular fractures were significantly more common in high-energy trauma mechanisms, which is consistent with patterns observed in adolescent and young adult populations [12,13]. The presence of these fractures necessitated surgical management in several cases, highlighting the need for a multidisciplinary approach and thorough imaging evaluation in high-energy injuries. Importantly, these injuries may not always be apparent on radiographs, even when serial radiographs are obtained, as in our study. This reinforces the value of advanced imaging, like CT and even MRI, in selected patients [13,17].

The proposed classification system helped distinguish trends by comparing with skeletal maturity and BMI. For instance, Type II and IV injuries were more frequently seen in older adolescents and those with elevated BMI, supporting prior findings that skeletal maturity and body mass are risk factors for more complex injury patterns [18]. Importantly, our classification system demonstrated a statistically significant association with skeletal maturity (p = 0.049), while no significant association was observed with BMI (p = 0.749). This suggests that classification type may partially reflect developmental stage and skeletal maturity, which are known to influence injury susceptibility and fracture patterns in pediatric trauma. However, the lack of association with BMI indicates that body habitus alone does not drive classification assignment, supporting the multifactorial nature of the system. That being said, while the observed association between skeletal maturity and classification type may reflect differences in injury mechanisms and fracture patterns across developmental stages, the small sample size limits the ability to draw definitive conclusions regarding these relationships.

From a predictive standpoint, this system may allow clinicians to have a structured framework for describing injury patterns and generating hypotheses regarding factors associated with treatment complexity and clinical outcomes before advanced imaging is even obtained [19]. Future prospective validation could establish thresholds within each classification tier to predict specific outcomes, such as the probability of requiring surgical fixation or the risk of AVN development. Additional review is also needed to clarify its inter-observer and intra-observer reliability, which is currently limited by the cohort's small sample size. As this classification system is further refined and expanded with additional data, it has the potential to improve modeling of risk factors, such as growth plate status, and influence the likelihood and nature of pediatric hip dislocations. Ultimately, such a framework could serve as a valuable educational tool for pediatric healthcare providers and caregivers, enhancing early recognition and appropriate management of these injuries.

As with all retrospective studies, ours too has limitations that must be acknowledged. First is our small sample size (N = 34). This greatly limits the generalizability of our subgroup analysis and can be associated with factors such as the lack of a statistically significant correlation between time to reduction and complications. That being said, advancements in surgical techniques, imaging, and perioperative care may have mitigated some of the negative effects historically associated with delayed treatment. Second, there may be selection bias due to the exclusion of transferred patients with incomplete data, missed patients for whom the EMR query failed to capture (in part due to documentation), and reporting bias in complications due to those who were lost to follow-up.

Third, although the overall incidence of severe complications such as AVN (N = 3) and infection (N = 1) in our cohort is low, longer-term follow-up is generally required to fully characterize delayed complications, including chondrolysis or late-onset osteonecrosis. While our study had a minimum follow-up of six months, this duration may be insufficient to capture delayed-onset complications. To address this limitation, we performed a secondary subset analysis using the same variables, restricted to patients with a minimum of one year of follow-up, as shown in Table 5. Despite the smaller sample size in this subgroup, the incidence of complications remained low, and time to reduction continued to demonstrate no association with adverse outcomes. This analysis demonstrates that our findings are not driven by shorter follow-up durations and remain consistent even among patients with extended clinical surveillance.

Another limitation is that our classification system has not yet undergone external validation; thus, further work is needed to assess its predictive value for long-term outcomes and treatment planning. Finally, although we attempted to control confounding variables, the complexity of trauma care and variation in treatment protocols between emergency and surgical providers may have had unforeseen effects on outcomes.

We will conclude by detailing a case example of using our classification system for the management of a pediatric case. Figure 1 illustrates injury radiographs and a CT scan of the left hip dislocation in a 14-year-old female patient, Risser 4, after a fall.

**Figure 1:**
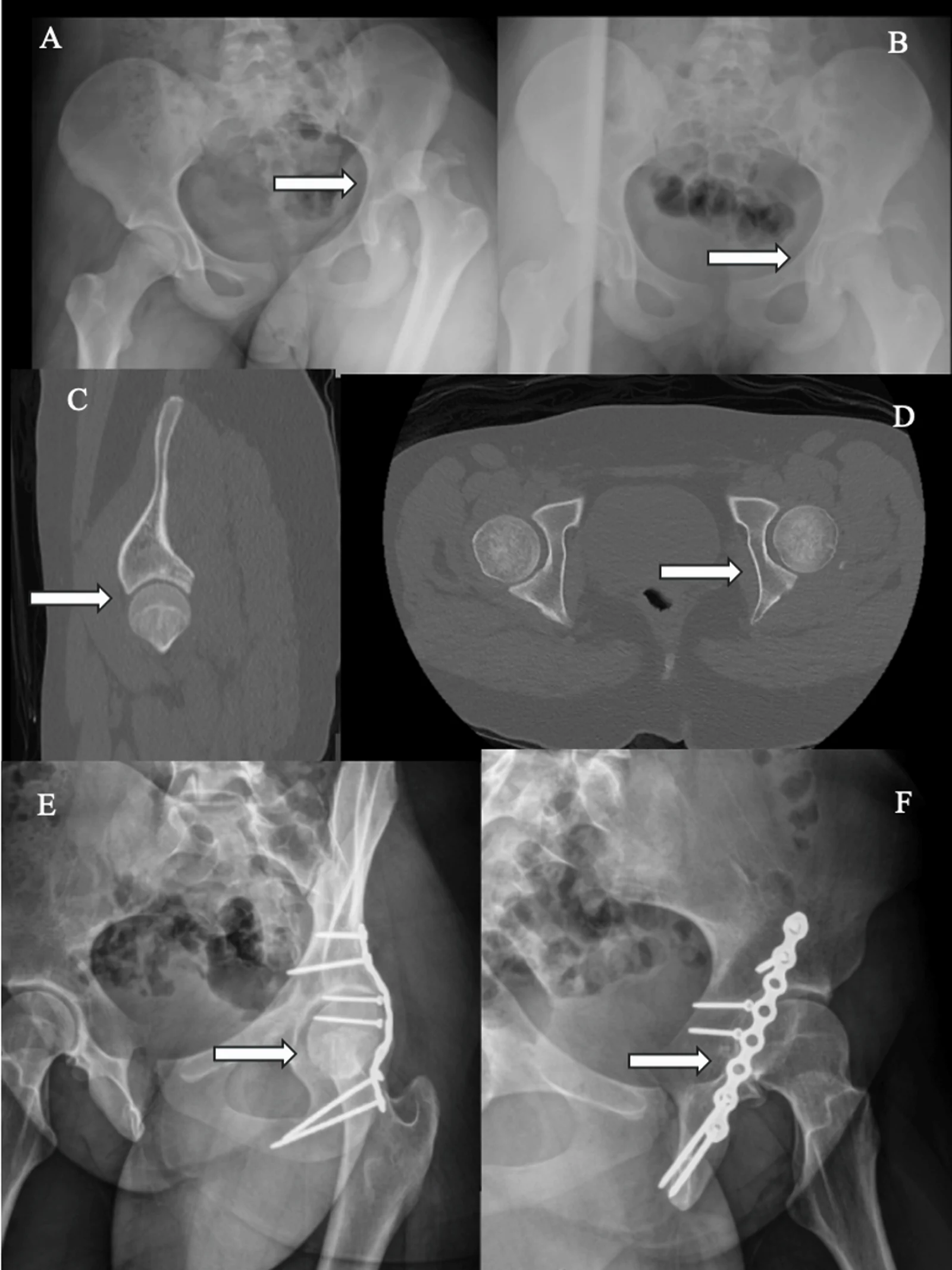
Dislocation radiograph (as labeled by arrow) in (A) and post-reduction radiograph (as labeled by arrow in (B)); sagittal and axial CT scan of the hip after reduction confirmed by arrows (C, D). Postoperative radiographs (E, F) with arrows demonstrating intact reduction and hardware.

Her BMI was in the 83rd percentile and decreased in the ED after four hours; post-reduction X-rays are shown below. Given her mechanism and injury, she falls into the Type II classification, as predicted by our findings. This low-energy, complex mechanism was associated with a posterior wall acetabular fracture-dislocation, which was managed surgically. This example demonstrates how our system can not only classify injury type but also anticipate the need for surgical intervention, inform rehabilitation intensity, and guide follow-up frequency based on risk stratification. As we build upon this classification system, we can further develop its validity and use it to guide future management of rare but important pediatric hip dislocations.

## Conclusions

In conclusion, our study highlights the continued relevance of mechanisms and associated injury patterns in pediatric hip dislocations, while challenging the primacy of time to reduction as the dominant predictor of complications. Our novel classification system offers a pediatric-specific approach to risk stratification, though it requires further prospective validation with studies of larger sample sizes. These findings reinforce the need for tailored pediatric trauma systems and longitudinal registries to track long-term outcomes and refine clinical guidelines for this rare but high-stakes injury.
